# Development and Pilot Testing of a Comprehensive Mobile Application to Assist Cell Count Determination During Peripheral Smear and Bone Marrow Examination

**DOI:** 10.7759/cureus.49597

**Published:** 2023-11-28

**Authors:** Arpit Gupta, Meyyappa Devan Rajagopal, Karthik Balajee Laksham

**Affiliations:** 1 Pathology, Jawaharlal Institute of Postgraduate Medical Education and Research, Karaikal, IND; 2 Pathology, All India Institute of Medical Sciences, Madurai, IND; 3 Community Medicine, Jawaharlal Institute of Postgraduate Medical Education and Research, Karaikal, IND

**Keywords:** malarial parasite quantification, differential count, bone marrow examination, reticulocyte count, platelet count, leucocyte count, hematology, mobile applications

## Abstract

Background: In the modern era of complete blood count analysis, manual differential count is performed whenever 'flags' are generated by an automated hematology analyzer. Traditionally, tally counters with five or eight keys are used for manual differential count. A few mobile applications are available to perform this task; however, the application features and cell representation are limited.

Objectives: The primary objective of our study was to develop an indigenous, comprehensive mobile application to assist with manual blood cell differential count. The secondary objective was to measure the usability of a newly developed application among undergraduate medical students.

Materials and methods: A new mobile application was developed using a Java development kit, Version 11.0.13 (Oracle Corporation, Austin, USA) in Android Studio Dolphin (2021.3.1) (Google, California, USA). The application content was validated by three pathologists with more than five years of experience. The app's usability was tested among 60 participants using a validated mHealth App Usability Questionnaire (MAUQ). The questionnaire had 18 items covering three domains: ease of use, interface & satisfaction, and usefulness.

Results: The newly developed application supports peripheral smear WBC differential count, platelet count, reticulocyte count, malaria parasite quantification, and bone marrow differential count. During usability testing, the app was easy to use in 95% (57/60) of participants, time-efficient in 91.7% (55/60), and helpful for healthcare practice learning in 96.7% (58/60). The total mean score was 6.11, indicating high usability.

Conclusion: A comprehensive mobile application to assist manual differential count with adequate cell representation was developed. The mobile application was easy to use, time-efficient, and valuable among the study participants.

## Introduction

Complete blood count (CBC) analysis plays a vital role in the initial screening of hematological disorders. It provides a rapid clue to the underlying pathology and points to the vital diagnostic tests that must be performed [1]. It is also one of the most frequently performed investigations in pathology laboratories, accounting for nearly 70% of the workload [2]. Automated hematology analyzer has revolutionized the process of CBC analysis by providing rapid results using advanced technologies like flow cytometry, electrical impedance, light scatter, fluorescent scatter, or cytochemistry [3]. Few analyzers like CellaVision are also capable of identifying and counting cells automatically after making peripheral smears [4]. Despite these advancements, manual smear examination is required whenever the auto-analyzer generates flags [5]. The manual smear review rate ranges from 10% to 50% in different laboratories, depending on the clinical population and local guidelines for smear review [6,7].

Manual tally counters with five or eight keys are widely used to assist in the manual enumeration of cells. However, these manual tally counters lack editing capacity, must be procured sufficiently for each laboratory setting, and are relatively expensive [8]. Mobile applications (apps) are widely used in the field of medicine to assist healthcare providers as well as patients with manual work [9-11]. Digital tally counters in mobile apps can overcome most of the drawbacks of manual tally counters. It has also shown a high correlation coefficient (r ≥ 0.899) in determining the manual differential count. Though few mobile apps are currently available to assist with manual differential count, the app features and representation of the cell population are limited [8]. The present study was undertaken to develop an indigenous, comprehensive mobile app to assist with manual differential blood cell count. The secondary objective was to measure the usability of the newly developed app among undergraduate medical students.

## Materials and methods

Study design

This was a prospective, cross-sectional, observational, pilot study conducted from August 2022 to December 2022 at Jawaharlal Institute of Postgraduate Medical Education and Research (JIPMER), Karaikal, India. The study was executed in two phases. In the first phase, an indigenous comprehensive mobile app for manual cell count on smear was developed, henceforth referred to as 'Hematology tally counter'. In the second phase, the app's usability was assessed among undergraduate medical students.

Phase I - 'Hematology tally counter' app development: A preliminary search on the availability and features of the existing hematology tally counter app was performed on the Google Play Store. The search was conducted on August 2022 using the keywords 'hematology', 'blood', 'cell', 'WBC', 'differential', 'tally', 'counter', and 'calculator'. Seven relevant mobile apps were identified, and their features were reviewed. The peripheral smear tally counter option was available in all seven apps; however, the cell representation was limited to five apps. The ‘Bone marrow tally counter’ option was available in two apps, the ‘reticulocyte counter’ in three apps, and the ‘platelet counter’ in two apps. None of the apps had an option for malaria parasite quantification. An all-in-one app encompassing the relevant features to support manual smear examination was lacking. Therefore, a comprehensive list of tally counters required during manual smear examination and the cell representation for each category was prepared after considering the limitations of existing apps. The newly prepared list includes peripheral smear WBC differential count, platelet count, reticulocyte count, malaria parasite quantification in the thick smear, malaria parasite quantification in the thin smear, and bone marrow differential count.

The user flow diagram, design, list of cells, cell images, cell description, and result interpretation for each tally counter was drafted in 'Microsoft Office Word'. A total of 18 cell images were captured from the six archived slides in the Department of Pathology. The app content was circulated among three pathologists with more than five years of experience for expert opinion. Each pathologist responded independently. The incorporation of 'Undo' and 'Others' keys in peripheral smear WBC differential count and bone marrow differential count was suggested by the experts. The app content was subjected to minor revision by incorporating the above changes. The final content and the user flow diagram were shared with the Information technology (IT) expert for app development. The app was developed using a Java development kit, Version 11.0.13 (Oracle Corporation, Austin, USA) in Android Studio Dolphin (2021.3.1) (Google, California, USA), an integrated open-source application development environment for the Android operating system. A trial run was performed by installing the newly developed app on an Android mobile phone. The practical difficulties, technical errors, and bugs were identified and rectified with the help of an IT expert.

Phase II - Usability testing: A cross-sectional study among undergraduate medical students assessed the user experience of the app. Students of both genders using Android mobile phones in second and third-year MBBS (Bachelor of Medicine and Bachelor of Surgery) were included in the study. Students who had not attended the briefing session and those who were on leave on the day of the survey were excluded. Faulkner L [12] has shown that 50 participants can identify a minimum of 98% of usability problems, and the detection rate increases with an increase in sample size. Approximately 60 students undergo MBBS each year in the Institute. Therefore, a sample size of 60 was included in the survey. A brief study overview was presented to all second and third-year MBBS students. Those willing to participate in the study were invited to the Department of Pathology on a specified date and time, out of regular class hours, with prior approval from the competent authority. Convenient sampling was performed by recruiting the first 60 willing participants who turned up on the survey day. Informed signed consent was obtained from all eligible participants. Confidentiality of data was maintained throughout the study. There was a briefing session on the morphology and manual enumeration of blood cells on smear. This was a review session and ensured that all participants received the same information. A link to the newly developed app was circulated to all participants, and the app was installed on their Android mobile phones. One smear for each test was provided to individual participants to perform the various manual counts using the app. After performing all six tests, the participants responded to the usability questionnaire. A validated mHealth App Usability Questionnaire (MAUQ) developed for standalone mobile health apps used by healthcare professionals was used for usability testing [13]. The questionnaire had 18 items covering three domains: ease of use, interface & satisfaction, and usefulness. Response for each item was scored on a scale of one to seven (1-Strongly disagree, 2-disagree, 3-somewhat disagree, 4-neither agree nor disagree, 5-somewhat agree, 6-agree & 7-Strongly agree). The total and average of the responses for all statements were calculated to determine the app's usability. The higher the score, the higher the usability of the app. The questionnaire also had open-ended suggestions for further improvement of the app. The investigators and experts critically analyzed the responses. The responses found to be crucial and relevant for better app functioning were incorporated into the final version. We also reviewed our newly developed app using the Mobile app development and assessment guide (MAG). MAG provides a validated checklist for identifying quality mHealth-related apps [14].

Statistical analysis

Categorical variables were expressed as percentages and continuous variables were expressed as mean (± Standard deviation). For each questionnaire statement, the mean response score was determined. The user experience was assessed based on the mean score. The higher the mean score, the higher the usability of the application feature. Data was analyzed using Epi Info 7.0 statistical software (developed by the Centre for Disease Control and Prevention, Atlanta, USA).

Research ethics

The study was approved by the JIPMER Undergraduate Research Monitoring Committee, Puducherry, India (JIP/UGRMC/NON-AWARD/2022/22, dated 18.07.2022) and Institute Ethics Committee (JIP/IEC-OS/2022/290, dated 30.08.2022). Informed signed consent was obtained from all participants. All methods were performed in accordance with the Declaration of Helsinki. Participants’ details were de-identified so that they may not be identified in any way. The reporting of this study conforms to Strengthening the Reporting of Observational Studies in Epidemiology (STROBE) guidelines [15].

## Results

**Figure 1:**
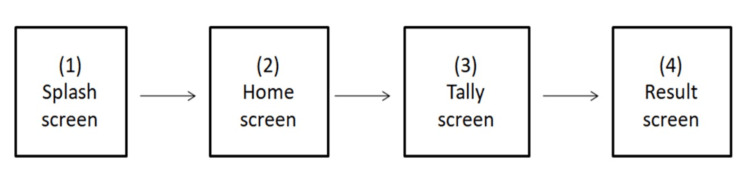
User flow diagram

**Figure 2:**
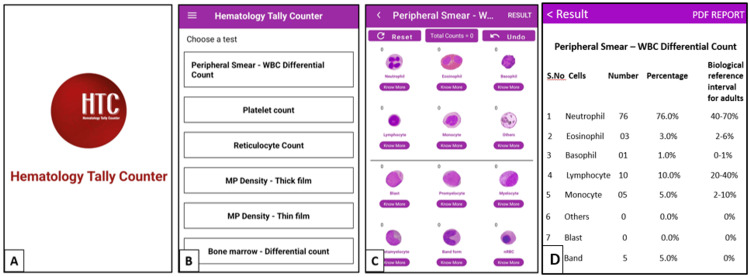
Screenshot of the app for peripheral smear WBC differential count A-Splash screen, B-Home screen, C-Tally screen, D-Result Screen

In phase 1, an Android mobile application having all relevant features for manual blood cell count on smears with adequate representation of cells was developed. The application supports peripheral smear WBC differential count, platelet count, reticulocyte count, malaria parasite quantification, and bone marrow differential count. The default maximum possible differential cell count using the app is 500. The overall navigation sequence of the app is provided in Figure 1, and the corresponding screenshot of the app for peripheral smear WBC differential count is provided in Figure 2.

The list of the cells/parameters included in each tally screen is provided in Table 1. An end user can choose the required test available on the home screen. This will take them to the corresponding tally screen, which has representative cell images. The user can start counting the cells by clicking the cell images. For novice users, a description of each cell is provided in the 'Know more' section beneath each image to facilitate cell identification. The app provides an instruction guide wherever required to facilitate quick learning of operating procedures and navigation sequences. The app is equipped with a sound and vibration facility for each click. It also provides an alert message after every 100-cell count. The user can turn on or turn off the sound and vibration settings depending on their preference. Even though the sound and vibration settings are turned off, the app provides an alert message at every 100-cell count. The app also provides an 'Undo' option to cancel the previously entered values and a 'reset' option to begin a new counting. After completing the counting process, the summary of findings can be generated by clicking the 'Result' button.

**Table 1:** List of cells/parameters in each tally counter screen

S.No	Tally screen	List of cells/parameters included
1	Peripheral smear - WBC differential count	Neutrophil, Eosinophil, Basophil, Lymphocyte, Monocyte, Others, Blast, Promyelocyte, Myelocyte, Metamyelocyte, Band form, nucleated red blood cell (nRBC).
2	Platelet count	Platelets in fields 1 to 10, Multiplying factor.
3	Reticulocyte count	Field count, Reticulocyte, Patient's hematocrit, Average RBC per field
4	Malaria parasite quantification – Thick film	Malaria parasite (Asexual form), WBC.
5	Malaria parasite quantification – Thin film	Malaria parasite (Asexual form), Field count, Average RBC per field.
6	Bone marrow differential count	Blast, Promyelocyte, Myelocyte, Metamyelocyte, Band form, Neutrophil, Eosinophil, Basophil, Lymphocyte, Monocyte, Erythroid, Megakaryocyte, Plasma cell, Macrophage, Others

In phase 2, usability testing was performed among 60 participants. There were 29 male (48%) and 31 female participants (52%). Fifty-five (92%) were from the second year, and five participants (8%) were from third-year MBBS. Each participant used the app and responded to the questionnaire. The response rate was 100%. Three domains were assessed using 18 questionnaire statements. The overall possible score ranged from 18 to 126. The overall total and average scores for the responses received were 110 and 6.11, respectively. The mean scores for five items in the 'Ease of use' domain ranged from 5.82 (±1.2) to 6.4 (±0.84). The mean scores of seven items in the 'Interface & Satisfaction' domain ranged from 5.85 (±0.91) to 6.33 (±0.75). The mean scores of six items in the 'Usefulness' domain ranged from 5.88 (±0.97) to 6.28 (±0.78). Among the participants, 95% (57/60) concurred (somewhat agree, agree & strongly agree) that the app was easy to use, 91.7% (55/60) concurred that it was efficient in time, 95% concurred that the app was easy to learn and 96.7% (58/60) concurred that it was helpful for healthcare practice/learning. The summary of responses for each domain and item is provided in Table 2.

**Table 2:** Summary of responses for usability testing

Domain	Item	Negative response (Score 1,2 &3)	Neutral response (Score 4)	Positive response (Score 5, 6 & 7)	Mean score (S.D)
		n (%)	n (%)	n (%)	
Ease of use	1. The app was easy to use	0 (0)	3 (5)	57 (95)	6.38 (±0.84)
	2. It was easy for me to learn to use the app	1 (1.7)	2 (3.3)	57 (95)	6.4 (±0.84)
	3. The navigation was consistent when moving between screens	1 (1.7)	5 (8.3)	54 (90)	6.15 (±0.94)
	4. The interface of the app allowed me to use all the functions offered by the app	1 (1.7)	5 (8.3)	54 (90)	6.18 (±0.94)
	5. Whenever I made a mistake using the app, I could recover easily and quickly.	6 (10)	2 (3.3)	52 (86.7)	5.82 (±1.2)
Interface & Satisfaction	6. I like the interface of the app.	0 (0)	5 (8.3)	55 (91.7)	6.05 (±0.83)
	7. The information in the app was well organized, so I could easily find the information I needed.	0 (0)	6 (10)	54 (90)	6.08 (±0.88)
	8. The app adequately acknowledged and provided information to let me know the progress of my action.	2 (3.3)	3 (5)	55 (91.7)	5.85 (±0.91)
	9. I feel comfortable using this app	2 (3.3)	3 (5)	55 (91.7)	6.15 (±0.96)
	10. The amount of time involved in using this app has been fitting for me.	0 (0)	5 (8.3)	55 (91.7)	6.1 (±0.89)
	11. I would use this app again if required.	0 (0)	1 (1.7)	59 (98.3)	6.33 (±0.75)
	12. Overall, I am satisfied with this app	0 (0)	3 (5)	57 (95)	6.23 (±0.84)
Usefulness	13. The app would be helpful for my healthcare practice/learning.	0 (0)	2 (3.3)	58 (96.7)	6.28 (±0.78)
	14. The app improved my ability to deliver healthcare services / perform the given task.	0 (0)	5 (8.3)	55 (91.7)	6.1 (±0.89)
	15. The app helped me to manage the given task effectively by providing an adequate representation of cells for each test/counter.	1 (1.7)	5 (8.3)	54 (90)	6.07 (±0.96)
	The app has all the functions and capabilities I expected.	2 (3.3)	3 (5)	55 (91.7)	5.93 (±1.01)
	I could use the app even when the Internet connection was poor or not available.	0 (0)	6 (10)	54 (90)	5.88 (±0.97)
	This app provides an acceptable way to deliver healthcare services.	1 (1.7)	5 (8.3)	54 (90)	6.02 (±0.96)
Overall mean score					6.11

We also received suggestions from the participants for further app improvement through open-ended questions. The participants' suggestions were critically analyzed, and those found to be appropriate were included in the app's final version (Table 3). The quality review of our newly developed app was performed using a validated checklist from MAG [14]. The app features fulfilled the relevant criteria for usability, privacy, security, appropriateness/suitability, transparency, content, safety, technical support/update, and technology.

**Table 3:** List of suggestions from participants included in the final version of the application

S.No	Study participant's suggestion	Action Taken
1.	Include biological reference interval in 'Result.'	Biological reference interval for adults included.
2.	The app has too much pink color. Some colors may be changed	App color changed to purple and white
3.	The 'Reset' key can be placed away from the 'Undo' key	The 'Reset' key shifted to the extreme left and the 'Undo' key to the extreme right
4.	Include microscopic images of cells in the 'Know more' section	Included
5.	Provide a 'zoom in' option for the 'Know more' section	Included

## Discussion

CBC, peripheral smear, and bone marrow examination play a crucial role in evaluating and diagnosing hematological disorders. Automated hematology analyzers are widely used for CBC analysis to provide rapid, reliable, and accurate results [16]. However, analyzers cannot precisely identify abnormal cells, immature myeloid cells, reactive lymphocytes, large platelets, and platelet clumps, and this successively results in 'flag' generation. In such a scenario, a manual smear review is performed for reliable identification and differentiation of cells [17]. Tabletop tally counters with five or eight keys are traditionally used to assist manual differential counting. The advent of IT has revolutionized the practice of diagnostic pathology as well as patient care by providing digital platforms to assist day-to-day work [18-20]. Students, laboratory technicians, and pathologists are routinely using portable electronic devices like mobile phones. Therefore, providing a digital tally counter as a mobile app would benefit the present tech-savvy students and laboratory professionals. 

Thurman et al. [8] provided the first systematic evidence of the performance of an app-based digital tally counter. They showed excellent agreement (r ≥0.899) between the app-based digital counter and standard tabletop analog counter for assisting manual differential count. App-based tally counters are relatively more portable than physical tabletop tally counters. Moreover, the data can be edited and easily transferred to other devices, avoiding errors due to manual entry. Few mobile apps are available to perform manual differential count; however, the application features and representation of the cell population are limited. Our newly developed 'Hematology tally counter' app provides a comprehensive list of tally counters required during manual smear examination. Apart from peripheral smear WBC differential count and bone marrow differential count, our app has multiple other features, which include platelet count, reticulocyte count, malaria parasite quantification in thick film, and malaria parasite quantification in thin film, thus providing the entire range of features to end users within a single app.

Evaluating the usability of a mobile app is an essential step before releasing it to end users. There are multiple ways to test the app's usability, one of which is to use a feedback questionnaire. We have used a validated questionnaire (MAUQ) designed for standalone mobile apps for health care providers. MAUQ has shown high reliability with an overall Cronbach alpha of 0.914. It also has strong construct and criterion validity and high internal consistency [13]. MAUQ has been used in multiple other studies for usability testing of newly developed mobile health apps [21-23]. The comprehensive list of tests available in our app is supported by the response offered by the study participants during usability testing, where 91.7% (55/60) concurred that the app had all expected functions and capabilities they expected it to have with a mean score of 5.93 (±1.01). Moreover, 90% (54/60) of participants concurred that the representation of the cell population for each counter is adequate, with a mean score of 6.07 (±0.96). The mean score for various other items assessed under ease of use, interface & satisfaction, and usefulness was also satisfactory. The total mean score was 6.11 indicating high overall usability of the newly developed app.

The app was developed by involving experts in the field, and suitable technical features were incorporated. The app revealed a high usability score (overall mean score - 6.11) and fulfilled the essential criteria listed in the MAG checklist. Therefore, this app would potentially benefit students, laboratory technicians, and pathologists during manual smear examinations by providing an all-in-one user-friendly interface. Mobile applications are portable, simple, and easy to use, with an added advantage over conventional key counters. A few minor modifications in the design and display of contents were made in the final version based on user feedback (Table 3).

Limitations

This being a pilot study, usability testing was performed among undergraduate medical students, limiting the findings' generalizability. A similar usability testing among laboratory technicians and pathologists with a comparison to a similar method may give a broader perspective on the usability of our newly developed app. Though the study participants had used manual tally counters during their training program for performing differential counts, a comparison between the app and manual tally counters was not performed in this study.

## Conclusions

Integrating mobile technology into healthcare not only streamlines the workflow but also enhances accessibility, marking a substantial step forward in the convergence of technology and medical practices. Manual smear examination plays a crucial role in determining blood cell count whenever flags are generated by automated hematology analyzers. In this study, a comprehensive mobile application with an adequate representation of blood cells for determining manual differential count was developed. The app was found to be easy to use, time-efficient, and valuable among the study participants.
